# Autonomous Sensors for Measuring Continuously the Moisture and Salinity of a Porous Medium

**DOI:** 10.3390/s17051094

**Published:** 2017-05-11

**Authors:** Xavier Chavanne, Jean-Pierre Frangi

**Affiliations:** Institut de Physique du Globe de Paris, University Paris Diderot, Sorbonne Paris Cité, UMR 7154 CNRS, Case postale 7011-F75205 Paris CEDEX 13, France; frangi@ipgp.fr

**Keywords:** profile of soil moisture, salinity and temperature, permittivity measurement, self-balanced bridge, electrode admittance, sample volume, autonomous sensors, wireless network, vadose zone

## Abstract

The article describes a new field sensor to monitor continuously in situ moisture and salinity of a porous medium via measurements of its dielectric permittivity, conductivity and temperature. It intends to overcome difficulties and biases encountered with sensors based on the same sensitivity principle. Permittivity and conductivity are determined simultaneously by a self-balanced bridge, which measures directly the admittance of sensor electrodes in medium. All electric biases are reduced and their residuals taken into account by a physical model of the instrument, calibrated against reference fluids. Geometry electrode is optimized to obtain a well representative sample of the medium. The sensor also permits acquiring a large amount of data at high frequency (six points every hour, and even more) and to access it rapidly, even in real time, owing to autonomy capabilities and wireless communication. Ongoing developments intend to simplify and standardize present sensors. Results of field trials of prototypes in different environments are presented.

## 1. Introduction

Continuous, in situ and, potentially, real-time monitoring of water content in a porous medium, like a soil, a compost or a silo, is in large demand for academic studies as well as economic activities. Sensors with such capabilities can be used to study plant growth in agronomy or regulate irrigation in agriculture. They allow for better modeling the interactions between climate and soil moisture of a catchment area in hydrology [1], but also to control ground stability under transport infrastructures, or concrete hardening process in civil engineering [2]. Academic studies would necessitate mapping soil moisture over a large area (from less to several km^2^) for long periods (several months) with high spatial and temporal resolution (down to 50 m between points and with an acquisition every 10 min.), preferably with vertical profile at each point, implying to work with a network of autonomous sensors [3]. Professionals would prefer user-friendly and rugged sensors. These would be able to cope with extreme media in terms of measurement such as compost or nutrient-rich greenhouse substrates. Both types of applications would need cost-effective sensors without compromising on their robustness and accuracy. They would have to measure intrinsic and representative quantities of the medium, related to its humidity, and readily available.

The standard method—sample weighting and drying in oven—provides directly the mass of water content, but is too destructive and time-consuming for continuous and in situ monitoring [4].

Various alternative techniques, indirect but better suited to above requirements, have been developed for measuring water content in a porous medium. On their own, each has benefits and limitations [1,5]. Among them, techniques based on medium permittivity have been extensively used, at least since the 1960s, for limited tests [6,7] and routinely in the field since 1980 [8]. They benefit from progress in electronic components and circuits, and now in information and communication technology (ICT). Dielectric permittivity of a medium measures its capacity to be polarized under the electric field E induced by sensor circuits. Electric dipoles of the medium tend to orientate along E against thermal diffusion, resulting in an equilibrium. Electric energy is stored in the process and returned when sensor excitation ceases. Relative dielectric permittivity $\varepsilon_{r}$ (medium permittivity normalized by vacuum permittivity $\varepsilon_{0}=8.854$ pF·m^−1^) varies from $\varepsilon_{r}=80.2$ for liquid water at 20 °C, due to its molecular dipoles, to only $\varepsilon_{r}=2$ to 4 for dry soils made of air and silicates (as well as bound water. However, the latter acts like ice, of which relative permittivity is about 3 [2]), which explains its interest. Presence of free charges in medium—in pore water but also on grain surfaces [9]—complicates the phenomenon. Under E, they not only produce dipoles at medium non-conductive interfaces, but also a transport current, which dissipates electric energy. Another mechanism is the delay of dipole response to sensor excitation, or dipole relaxation, due to molecular interactions and ion mobility. The effect of a simple relaxation is described by Debye’s relation using an intrinsic delay time or its inverse, a relaxation frequency [2]. Therefore, when operating with alternating fields at frequency *f*, permittivity depends on *f*. That of liquid water decreases from its value of 80.2 at 20 °C towards 5.6 when *f* increases, with half of the fall at the relaxation frequency 17 GHz [10]. Dissipation also occurs with relaxation and is maximal at the relaxation frequency while zero far from it. Relaxation of other forms of dipole in the medium occur at much lower frequencies, below about 10 MHz [2,11]. Dipole relaxations, along with current of charges, introduce a phase shift of the overall medium response to the electric excitation. Hence, the complex expression of permittivity ($j^{2}=-1$):

(1) $$\varepsilon_{r}=\varepsilon_{r}-j\;\frac{\sigma }{\varepsilon_{0}\;2\pi \;f}\;.$$

Real part $\varepsilon_{r}$ thus represents pure medium polarization dominated by molecular dipoles when frequency is in the range between about 10 MHz and 1 GHz. Imaginary part σ represents medium bulk conductivity associated with pore ion conductivity, frequency independent, and to loss due to dipole relaxations. Various models exist to relate these electric quantities to the physical ones, such as moisture and salinity, from an empirical correlation [8] to mixing equations [2] or to models based on the application of the electromagnetism theory at the grain scale [9]. Their description and merits are beyond the scope of this article. However, due to this intrinsic complexity of dielectric phenomena in a porous media, it is important for a sensor to be able to distinguish accurately between the two contributions of Equation (1), and to determine them in same conditions, in particular at the same frequency. Measurement of σ at a lower frequency can result in an overestimate of the ionic conductivity due to the then important contribution of relaxations of dipoles from charges in medium. It is all the more crucial when sensors are used to test and calibrate the models, working with controlled porous media. In addition, both permittivity and conductivity are often of interest for a final user (such as for monitoring nutriments or salinity in soils). Due to the influence of medium temperature, the latter will be equally required.

Furthermore, sensors introduce bias, all the more since neither measures directly the permittivity due to their technique itself. In particular, the dependence of the sensor output on circuit resistance (hence on the bulk conductivity σ of the medium) can make the permittivity determination incorrect [12,13,14]. In order to have a reliable and specific assessment of sensor performances, and because soils can not be considered as standards for this purpose, they should provide permittivity readings and be calibrated against homogeneous fluids of known permittivity [13,15]. Besides tending to comply with this exigence, some manufacturers recently delivered products able to measure σ as well. On the other hand, measurements are very often performed with a technique different from the one to determine $\varepsilon_{r}$, and therefore not in the same conditions. Permittivity-based instruments produce in the medium under study electromagnetic fields propagating along a direction *z*, which corresponds to the axis of sensor electrodes or guide. Fields are solutions of Maxwell’s electromagnetic equations (see Introduction in [11,16]) and present the general form:

A(r)ph(2πz/λ−2πft),

where *A* is the field amplitude at point r in the medium, ph the field phase and λ their electromagnetic wavelength. Both *A* and λ depend on $\varepsilon_{r}$. The expression of λ is:

(2) λ=cfRe(εr)≈cfεr,

where *c* is the velocity of electromagnetic waves in vacuum (*c* approximately 0.30 Gm·s^−1^). Re(X) is the real part of the complex *X*. $\varepsilon_{r}$ is given by Equation (1).

The field wavelength determines two types of instruments. For λ lower than the sensor length h∼10 cm, which corresponds to frequency *f* larger than about 100 MHz, electromagnetic propagation is the dominant phenomenon. An estimate of the real part $\varepsilon_{r}$ is usually based on Equation (2) and the measurement of λ or phase velocity f·λin the medium. The most common instrument in this category is the time domain reflectometry (TDR) sensor using step transverse waves propagating along a metallic guide in the medium [8,17]. The wavelength λ is deduced from the round-trip time of waves. One of the leading TDR manufacturers is Campbell Scientific, Inc. (Logan, UT, USA). It provides a large range of TDRs, which have evolved over time from expensive two-part instruments, only measuring permittivity, to a compact cheaper device able to give the three medium variables, i.e., $\varepsilon_{r}$, σ and temperature (sensors CS650/655). However, the latter sensor does it at a lower accuracy than its two-part parent tanks to much simpler electronics to detect travel time. Moreover, detection becomes difficult in media with large salinity due to the attenuation of the reflected signal, which increases with guide length, and due to the dependence of wavelength λ on σ [18]. The latter problem is partially solved by determining σ from the wave attenuation itself. Campbell Scientific provides the following accuracy for its recent field sensor CS655 (length of 12 cm, up to $\sigma =0.3\;S\cdot m^{-1}$) $\delta \sigma =\pm \left[50+5\%\;\sigma \right]\;\mu S\cdot {\mathrm{cm}}^{-1}$ and $\delta \varepsilon_{r}=\pm \left[0.8+3\%\;\varepsilon_{r}\right]$.

For wavelength λ larger than h∼10 cm, which means an operating frequency *f* below or close to 100 MHz, but still larger than 10 MHz, electromagnetic propagation becomes negligible. The field expression is approximately A(r)ph(2πft), and permittivity is extracted from the field amplitude. Electromagnetic phenomena are then described by the electric circuit theory, including inductive effects (Section 5.18 of [16]). Fields are converted into voltages and currents. The system made of sensor electrodes and medium is modeled by a capacitor of which capacitance is related to $\varepsilon_{r}$. Capacitance sensors currently available measure either the frequency of an electronic oscillator comprising a capacitor (e.g., the EnviroSCAN and TriSCAN probes marketed by Sentek Pty Ltd. (Stepney, Australia) [12]), or the root mean square of charging and discharging cycles of a capacitor (the probes commercialized by Decagon Devices Inc. (Pullman, WA, USA) [14]). In both cases, only a part of the capacitor dielectric is made of the medium to be analyzed. Hence, the sensor output is related to $\varepsilon_{r}$ thanks to semi—or totally—empirical relations [12,19]. Empirical relations require many parameters and carry the risk of errors for measurements far from those done during calibration. Recent devices, such as the products GS3 of Decagon Devices and TriSCAN of Sentek Pty, perform reading of conductivity σ to correct the signal for its effect. However, measurements are not performed with the same technique, in particular the same frequency *f*, as for $\varepsilon_{r}$. Accuracy of sensor GS3 is reported as (for $\sigma <1\;S\cdot m^{-1}$): δσ=±10%σ and $\delta \varepsilon_{r}=\pm 5\%\;\varepsilon_{r}$. Sentek Pty does not indicate the accuracy of its devices on $\varepsilon_{r}$ and σ (it gives directly soil water content and its salinity with reported uncertainties of ±0.003% vol. and ±8.06%, respectively, within 4–20% moisture and 0–4.9 mS/cm salinity ranges).

In the category of capacitance sensors, a technique is based on a wave propagation along a transmission line, usually a coaxial one, and its reflexion at the line end [20,21]. The electrodes located at the end of the line are produced due to impedance mismatch of a reflected wave, which interferes with the incident one to form a partially standing wave. From the complex coefficient of a wave reflexion, these sensors can deduce the impedance of the electrodes in medium, at least its capacitance. Among the field sensors using this method, the main instrument commercialized by Stevens Water Monitoring System Inc. (Portland, OR, USA), Hydra Probe, operates at 50 MHz and claims an accuracy δσ=±[0.02+2%σ] S·cm^−1^ and $\delta \varepsilon_{r}=\pm \left[0.2+1.5\%\;\varepsilon_{r}\right]$ (for electrode length of 6 cm, and up to σ=1.5 S·m^−1^). Devices of the company Delta-T Devices Ltd. (Cambridge, UK) operate at 100 MHz and only measure permittivity (high frequency as well as cable and electrode lengths probably impede a measurement of electrode resistance). Delta-T offers both a point sensor—ThetaProbe—and a profile sensor—PR2—made of different pairs of electrodes along a vertical cylinder, a geometry very similar to the product EnviroSCAN of Sentek Pty but with a different measurement technique. No uncertainty for permittivity is given.

Another potential source of bias is the low representativity of medium sampled by sensors due to soil disturbance during installation, in particular air gap and soil compaction around electrodes, and poor sensor design. The latter affects the spatial distribution of sensor sensitivity [22]. Too thin electrodes or contact with medium through a low permittivity material (access tube or coating) reduce this extent. In addition, annular electrodes along the same cylinder, like for Sentek and Delta-t profile sensors, reduce the sensitivity extent.

As regards sensor autonomy, because manufacturers mentioned above developed their products before the advent of new ICT, devices are usually connected by cable to a data-logger, which also supplies power. Data-loggers themselves are powered either by the mains electricity or by a lead battery. Recently, Campbell Scientific has commercialized an autonomous wireless sensor (CSW650). However, it only investigates the first centimeters of soil, as the electronic housing should be located above ground for battery access and data transfer. Their legacy data-loggers are still used to collect the sensor data thanks to a radio module connected to it. In addition to these developments, a scientific group had set up a wireless network of 600 sensors, based on Decagon products, to equip an observatory in Lower Rhine valley (Germany) and investigate the subcatchment with high resolution [3]. They relied on the IEEE’s Zigbee protocol communication (at carrier frequency 2.44 GHz). Sensors at an area point are connected to a radio end device just below the ground, in communication within 100 m range to routers, in communication themselves to a coordinator.

**Figure 1:**
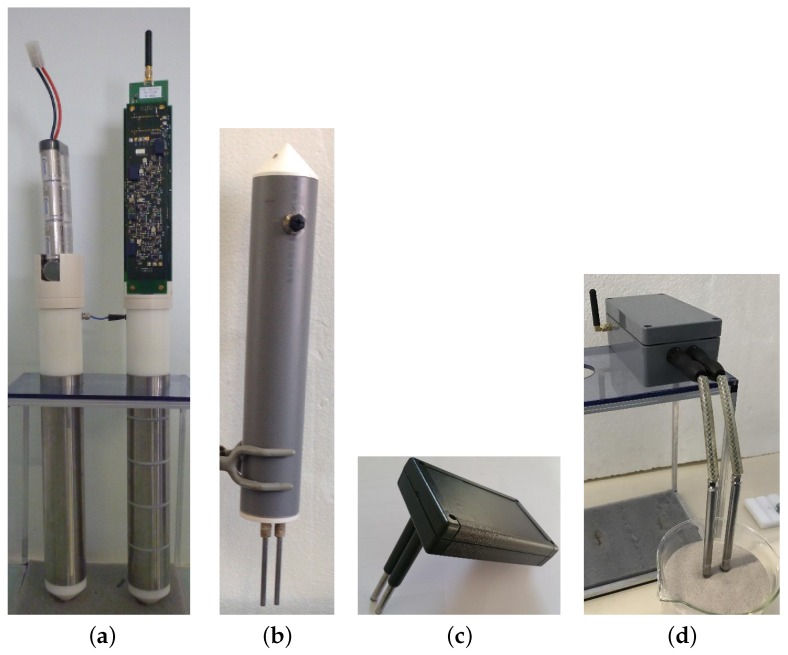
Prototypes developed in the laboratory with same physical principles. (**a**) multi-channel sensor for vertical profile owing to stacked electrodes; (**b**) one-channel sensor derived from the multi-channel one; (**c**) low cost sensor; and (**d**) one-channel simplified sensor.

For at least 10 years, our group has been pursuing a program—called HYMENET—of field admittance-meters to measure soil humidity, which intends to address all the fundamental and practical issues mentioned [11,15,23]. Figure 1 shows some realizations and ongoing developments of prototypes: a sophisticated multi-channel sensor for profiles, described in previous articles, a one-channel sensor using the same electronic circuits but with a more compact design, and, since 2016, various one-channel sensors for commercial purposes. They are all based on the same technique of measurement, namely, the self-balanced bridge. It is the most direct method to obtain the complex permittivity in Equation (1), that is, $\varepsilon_{r}$ and σ, simultaneously and in the same conditions. It features a high resolution, or large dynamic, while requiring low cost digital acquisition converters. The next section describes the physical modeling of the sensor and the calibration process to obtain accurate measurements, specifically in severe conditions, which were not covered previously. Resolution and accuracy are deduced. Developments to simplify the design and the quality control of sensors, in order to turn them into commercial products, are also stressed. The third section presents some results of field trials in various soils and different meteorological situations.

## 2. Materials and Methods

**Figure 2:**
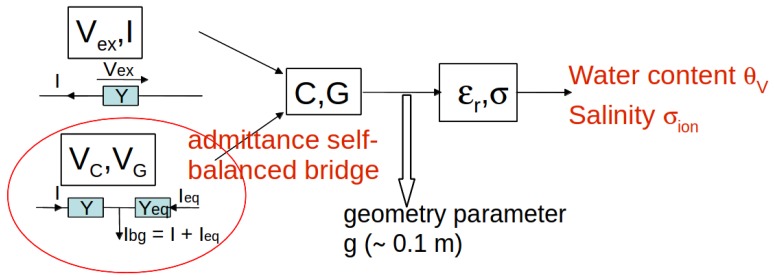
Successive conversions from sensor signals, bridge voltages $V_{G}$ and $V_{C}$, to the water content of a porous medium, $\theta_{v}$, and its salinity $\sigma_{ion}$. A previous system relied on a direct current-voltage measurement. *G* and *C* are, respectively, the capacitance and the conductance of the electrodes embedded in the medium, while $\varepsilon_{r}$ and σ are its apparent electric permittivity and conductivity.

Sensor electrodes embedded in the medium under study form a capacitor of which complex admittance is Y=G+jC2πf. *Y* is directly related to the apparent permittivity of the medium $\varepsilon_{r}$ in Equation (1) according to:

(3) $$\varepsilon_{r}=\frac{C}{\varepsilon_{0}\;g}-j\;\frac{G}{\varepsilon_{0}\;g\;2\pi \;f},$$

where *g* is a length depending on the geometry of electrodes. The complex admittance is measured by a self-balanced admittance bridge, which provides two direct voltages, $V_{G}$ and $V_{C}$. They are proportional in first order to *G* and *C*, respectively. Figure 2 shows schematically the different steps of the inverse process, in order to convert raw sensor signals into quantities of interest, i.e., medium water content and its salinity. Previous prototypes developed by our group measured the alternating voltage $v_{ex}$ applied between the electrodes and the resulting current *i*, and then deduced *Y* from Ohm’s law [15,23]. However, the operation requires A/D converters with high sampling frequency, at least 500 MHz given *f*, resulting in expensive components and low resolution. The bridge with its direct voltages does not suffer from these drawbacks. Physical modeling of the bridge and its connections to electrodes, along with the calibration procedure, in the case of media of low conductivity such as many soils, were presented in a recent article [11]. The present one describes a complete modeling of the sensor and a new calibration procedure in order to extend the range of measurement to media of high conductivity (such as compost or nutrient-rich soils), at least to σ=1 S·m^−1^ = 10 mS·cm^−1^.

The last step in Figure 2 relies on models independent of the instrument characteristics, except for its frequency, and therefore is not dealt with in this article. Moreover, they would require being tested and calibrated before use, owing to controlled porous media and sensors exempt of bias.

### 2.1. Physical Principle

#### 2.1.1. Self-Balanced Bridge

**Figure 3:**
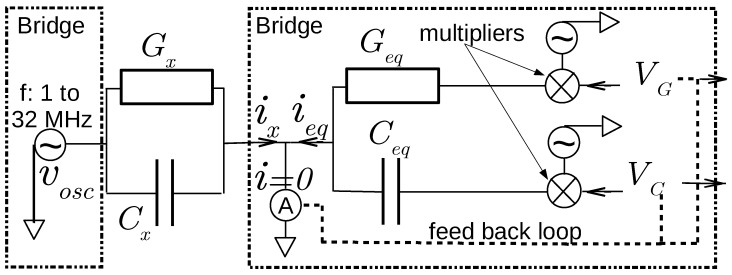
Schematic diagram of the self-balanced bridge. The current $i_{x}$ flowing through the admittance to be measured, $Y_{x}=G_{x}+j\;C_{x}\;2\pi \;f$, under bridge oscillator voltage $v_{osc}$ at frequency *f*, is balanced by the current $i_{eq}$. It is generated by the bridge owing to a feed back loop, multipliers and fixed components, conductance $G_{eq}$ and capacitance $C_{eq}$. The feed back loop provides the direct voltages $V_{G}$ and $V_{C}$.

Figure 3 presents the main features of a self-balanced bridge. It results from a collaboration with the firm CAPAAB (Chatenay-Malabry, France). An on-board oscillator provides a voltage $v_{osc}$ at a fixed frequency *f*—with a choice between 1 and 32 MHz to allow a study of its influence on measurements. The voltage $v_{osc}$ is applied to the admittance to be determined, $Y_{x}=G_{x}+j\;C_{x}\;2\pi \;f$, and it is used for bridge analog operations. A bridge is a zero method of measurement where the current flowing from the admittance is exactly canceled out by the bridge’s own current. It is automatically generated owing to a feedback loop, multipliers and fixed components, conductance $G_{eq}$ and capacitance $C_{eq}$. The feedback loop provides the direct voltages $V_{G}$ and $V_{C}$, each used as an input of a multiplier, along with the voltage $v_{osc}$. Hence, the direct voltages $V_{G}$ and $V_{C}$ relation to $Y_{x}$:

(4) $$\left\{\begin{array}{c} G_{x}=G_{eq}\;V_{G},\\ C_{x}=C_{eq}\;V_{C}. \end{array}\right.$$

Bridge sensitivity parameters $G_{eq}$ and $C_{eq}$ are fixed by specific components between 3.0 and 50 mS·V^−1^ for $G_{eq}$, and between 20 and 30 pF·V^−1^ for $C_{eq}$, depending on the application. Direct voltages are limited between −3.5 and 3.5 V, beyond which the bridge presents a saturation. Their resolution or reproducibility is about $\delta V_{Y}=\pm \left[0.2+0.5\%\;V_{Y}\right]$ in mV. Given the range of $V_{G}$ and $V_{C}$ and the sensitivity parameters, from Equations (3), (4) and (10), the sensor should be able to measure σ up to 1 S·m^−1^ (or *G* to 150 mS) and $\varepsilon_{r}$ up to 100 (as well as for *C* in pF). However, parasitic effects on the whole sensor must be included, specifically in Equation (4).

#### 2.1.2. Overall Instrument

Imperfections of the overall instrument result from inevitable electronic and electromagnetic phenomena in the case when a capacitance sensor is operating at high frequency. From bridge to electrodes, they are:

- Bridge offset drifts and interferences between *G* and *C* branches from non-ideal integrated circuits
- Parasitic admittance, in parallel, and impedance, in series, from leads to electrodes
- Electrode inductance

In addition, a multi-channel sensor presents interferences, or diaphony, between its channels.

They are first minimized by some actions described previously [11]: correction of bridge interferences owing to surface-mount potentiometers, addition of a capacitor in series to reduce the lead inductance, use of a relay at bridge input to perform an open-ended measurement, and then to cancel out bridge offsets and part of the admittance in parallel. These features are also intended to simplify the adjustments of sensors produced in series.

**Figure 4:**
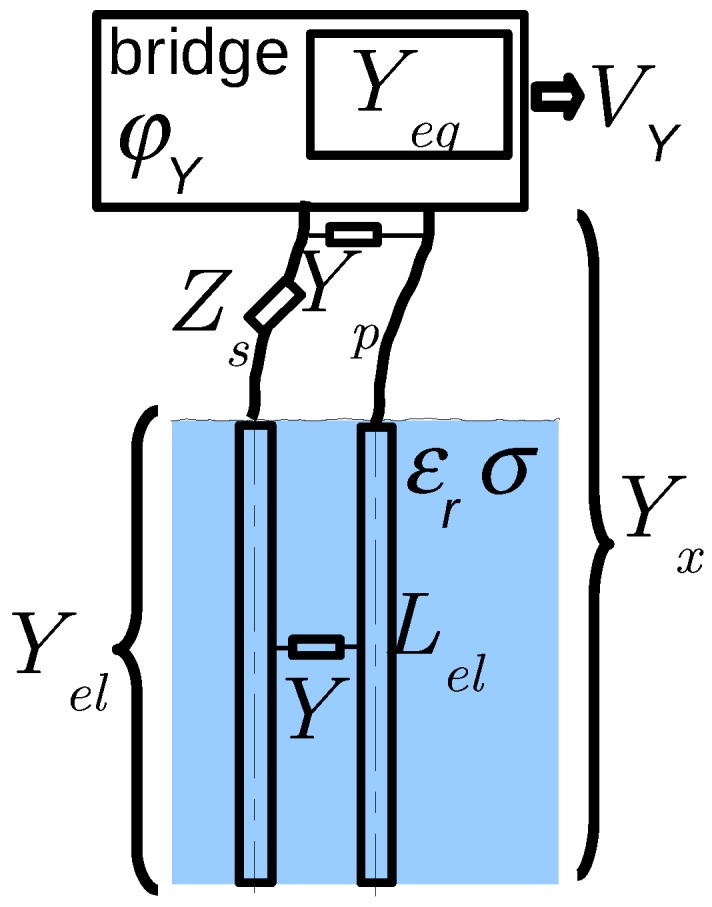
Instrument imperfections (in complex notation), as included in a physical model of the sensor: bridge interferences between *G* and *C* branches ($\varphi_{Y}$), parasitic admittance ($Y_{p}$) and impedance ($Z_{s}$) due to leads, and electrode inductance ($L_{el}$).

After this mechanical corrections, a model accounts for the residual differences from Equation (4), which require a set of six coefficients in addition of the two main parameters $G_{eq}$ and $C_{eq}2\pi \;f$ (see Figure 4):

- The bridge residual interferences $\varphi_{C/G}$ and $\varphi_{G/C}$ in rad
- The residual admittance, $Y_{p}=G_{p}+j\;C_{p}\;2\pi \;f$ in μS, and impedance, $Z_{s}=j\;L_{S}\;2\pi \;f+r_{S}$ in Ω
- Electrode inductance $L_{el}$

From their physical origin and for fixed components and dimensions, they are approximately known, which simplifies the calibration procedure. However, because of the dispersion of device characteristics and in the case of needing accurate measurements, a calibration is still required.

The last step of the model comprises the conversion of the total admittance $Y_{el}$ of electrodes into the admittance of interest *Y*. The conversion is given by the implicit equation [7]:

(5) $$Y_{el}=\frac{\tanh(\gamma )}{\gamma }\;Y,$$

where tanh is the hyperbolic tangent and γ is:

(6) $$\gamma^{2}=-a+j\;b,$$

or

(7) $$\gamma =\sqrt{-a/2+(\sqrt{a^{2}+b^{2}})/2}+j\;\sqrt{a/2+(\sqrt{a^{2}+b^{2}})/2},$$

with

(8) $$\left\{\begin{array}{c} a=L_{el}\;C\;{\left(2\pi f\right)}^{2}-R_{el}\;G,\\ b=L_{el}\;G\;2\pi f+R_{el}\;C\;2\pi f. \end{array}\right.$$

The electrode inductance $L_{el}$ and resistance $R_{el}$ only depends on their material and geometry, and therefore are known precisely. In the model, their values are fixed. The residual impedance $Z_{s}=j\;L_{S}\;2\pi \;f+r_{S}$, actually reduced to $L_{S}$ as $r_{S}$, is negligible, is adjusted owing to a calibration with homogeneous fluids.

### 2.2. Calibrations, Resolution and Accuracy

Previous articles only described a calibration process using reference capacitors $C_{ref}$ and resistors $R_{ref}$ placed between electrodes [11,23]. To determine finely the coefficients introduced above, in particular the residual inductance $L_{S}$ at fixed inductance $L_{el}$, a calibration with air and with water at different concentrations of KCl salt is necessary. Solution conductivity is measured by the conductivity meter C931 of the firm Consort (Turnhout, Belgium). For salinity below σ=1 S·m^−1^, the value of water real permittivity remains that of pure water [24], taking into account its variation with temperature.

**Figure 5:**
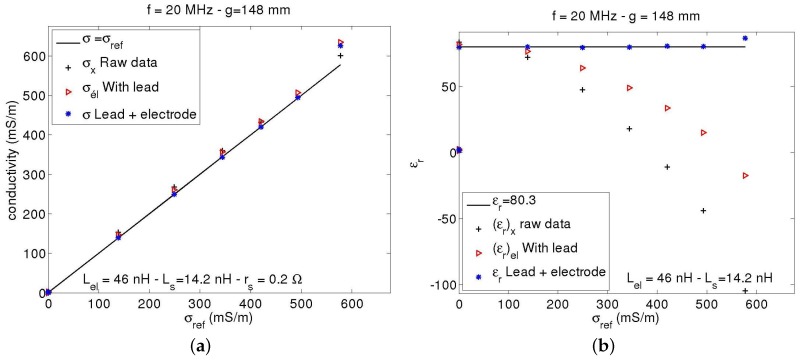
Calibration of a sensor with air and water at different concentrations of KCl salt as references (black lines). (**a**) sensor conductivity against measurements by the conductivity-meter C931 of the firm Consort (Belgium); (**b**) sensor permittivity against water permittivity. At high conductivity, adjustments depend tightly on inductances of leads and electrodes, the latter being fixed. The last point is affected by bridge voltage $V_{G}$ exceeding 3.5 V; sensor sensitivity was $G_{eq}=24$ mS·V^−1^.

Figure 5 presents the successive adjustments of sensor outputs to match reference values. They demonstrate the necessity of taking into account precisely lead and electrode inductances above σ=100 mS·m^−1^, such as in compost and nutrient-rich soils. Contribution of inductances in series with the capacitance *C* to the output $V_{C}$ becomes large, even more important than the contribution of *C*, leading to a negative value of permittivity without including their effects. For non-magnetic materials, these inductances, and therefore their effects, only depend on the geometry of leads or electrodes, namely, their length and the ratio between axis distance and diameter. For thin and distant pair of leads or electrodes, inductance increases by about 1 nH per mm of length, which is lower for close leads. In our case, owing to inductance offset by a capacitor in series, lead inductance is reduced to 14 nH, which corresponds to a total residual length of about 14 mm, or 7 mm along the pair. This amount is probably unavoidable, whatever the length of leads. Adjustments fit reference values within an uncertainty of about 1%. By increasing the value of $G_{eq}$—hence decreasing apparatus sensitivity—the range can be extended to σ=1 S·m^−1^, at the cost of reduced precision on the low conductivity end. The calibration procedure can be reduced to four measurements, one in air, another in de-ionised water and the last two in saline water at low and high conductivity (about σ=100 and 300 mS·m^−1^).

**Figure 6:**
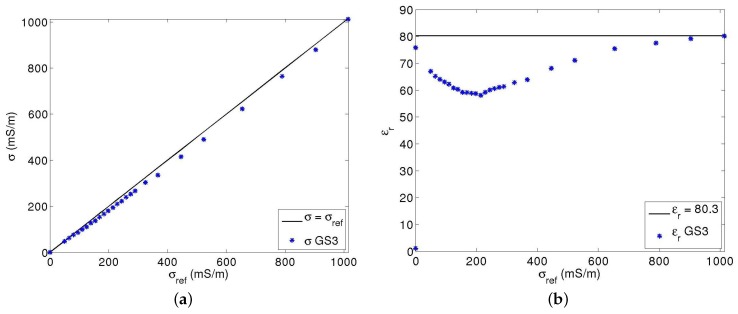
Comparison of the outputs of a GS3 Decagon sensor with known values using the same procedure as in Figure 5. (**a**) sensor conductivity against conductivity measurements by the conductivity meter C931; (**b**) sensor permittivity against air and water permittivity.

For comparison, the same procedure as in Figure 5 was applied to the GS3 Decagon sensor. Figure 6 shows the results. GS3 outputs include an unknown treatment to partially correct for the parasitic inductances. The device is intended to measure permittivity even beyond σ=1 S·m^−1^. If conductivity measurements appear satisfying, with a technique, however, operating probably at a lower frequency than for $\varepsilon_{r}$, the permittivity measurement is much more approximate at an intermediate salinity of water. Hydra Probe from Stevens Water Monitoring System suffers even more accurately from this problem [25]. For a salinity of an aqueous KCl solution at 140 mS·m^−1^, $\varepsilon_{r}$ decreases from 80 to 77. The trend accelerates at higher salinity, along with a dispersion of values between sensors. Conductivity measurements are also affected as the sensor uses, to deduce σ, the same input as for $\varepsilon_{r}$, namely, the complex reflection coefficient.

**Figure 7:**
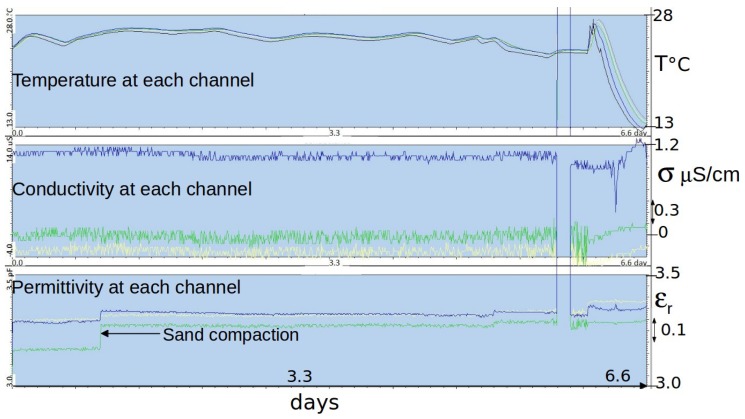
Time series of data from a multi-channel sensor in a dry sand. From data dispersion, the accuracy on permittivity and conductivity at low values is: $\varepsilon_{r}=3.30\pm 0.05$ and σ=0±1 μS·cm^−1^ (with sensor parameters $G_{eq}=5$ mS·V^−1^ and g=0.12 m). Compaction of sand by shaking its container reduces air presence close to electrodes, and, therefore, $\varepsilon_{r}$ differences between channels. Low temperature sensitivity of the sensor is demonstrated by varying external temperature at the end of the trial.

In addition to the calibration in air and water, a trial was carried out in a dry sand made of quartz at 99% to assess the accuracy of medium permittivity and conductivity measurements at low values. Dry sand can be considered as a homogeneous medium with no conductivity. Figure 7 shows records performed by a multi-channel sensor (sensitivity $G_{eq}=5$ mS·V^−1^ and geometry parameter g=0.12 m). Each channel had been calibrated independently using capacitors $C_{ref}$ and resistors $R_{ref}$. Uncertainty deduced from dispersions between channels and shift from the expected value for σ is about $\varepsilon_{r}=3.30\pm 0.05$ and σ=0±1 μS·cm^−1^. Resolution on $\varepsilon_{r}$ and σ deduced from those on bridge voltages $V_{G}$ and $V_{C},$ reported in Equations (3) and (4), would be $\delta \varepsilon_{r}=\pm 0.005$ and δσ=±0.1 μS·cm^−1^, i.e., lower than the actual uncertainty by a factor of about 10. The increase represents the difference between resolution and accuracy, which takes into account all of the parasitic effects.

Combining the results shown in Figure 5 and Figure 7, the overall uncertainty is about:

(9) $$\left\{\begin{array}{c} \delta \sigma =\pm \left[1-10+1\%\;\sigma \right]\;\mu S\cdot {\mathrm{cm}}^{-1},\\ \delta \varepsilon_{r}=\pm \left[0.05+1\%\;\varepsilon_{r}\right]. \end{array}\right.$$

Uncertainty on σ at low values depends on the parameter of sensitivity $G_{eq}$ increasing with it, with the trade-off to a larger range.

The trial in dry sand also intended to estimate the temperature sensitivity of electronic circuits by varying ambient temperature. Temperature induced variation on measured $\varepsilon_{r}$ and σ appears within the range of uncertainty. Thorough tests confirmed a low sensitivity on temperature at about 0.1%/°C. New bridge design is expected to bring it down to 0.02%/°C. This specification is important in order to interpret results of field trials such as those in Section 3.

### 2.3. Electrode Geometry and Sampling Volume

**Figure 8:**
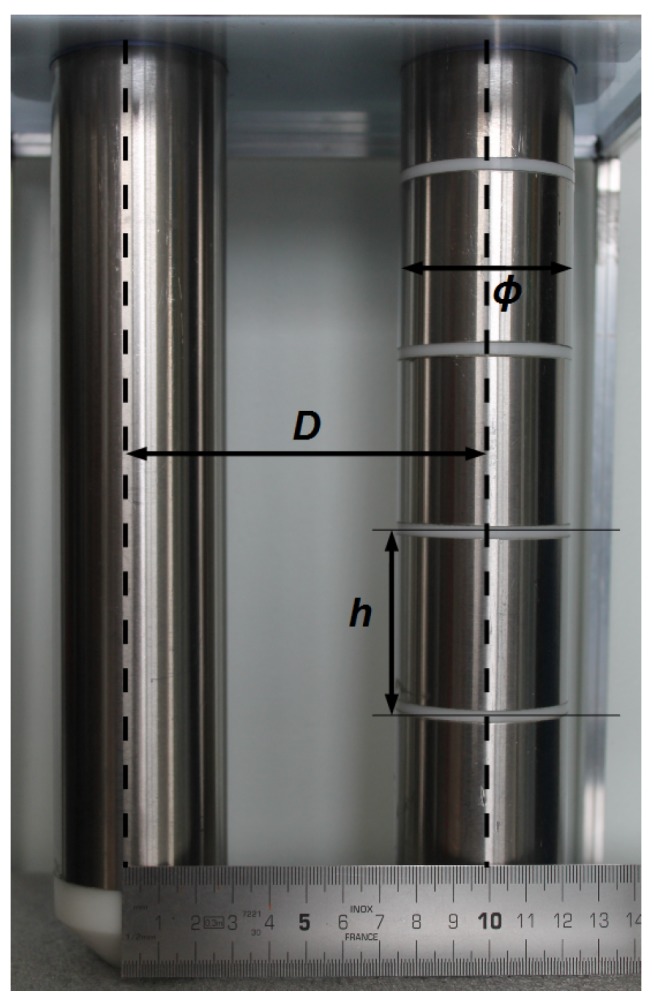
Electrodes of the multi-channel sensor in Figure 1a, with dimension symbols: Φ is the electrode diameter (Φ=50 mm here), *D* the distance between their axes (D=100 mm) and *h* their height (h=50 mm).

Conversion of electrode admittance *Y* determined by the bridge into the permittivity in Equation (3) requires the geometry parameter *g*. For a capacitor made of two parallel electrodes of height *h*, diameter Φ and axes distance or spacing *D* (see Figure 8), the parameter *g* is given by the theoretical relation [26]:

(10) $$g=\frac{\pi \;h}{\mathrm{arccosh}(D/\Phi )},$$

where arccosh is the inverse of the hyperbolic cosinus. The value of *g* varies from 0.12 m (sensor in Figure 1a) to 0.15 m (sensor in Figure 1b). The relation assumes no fringing effect. They are either eliminated owing to a guard at each end, like in the case of multi-channel sensors [11], or taken into account by a correcting factor close to 1. The factor depends only on electrode dimensions, namely, the ratios h/D and α=Φ/D. Numerical simulations, along with a laboratory validation, show that the discrepancy from Equation (10) is mostly dependent on the ratio h/D, and tends to zero as it increases (factor of 1.06 at h/D=4).

**Figure 9:**
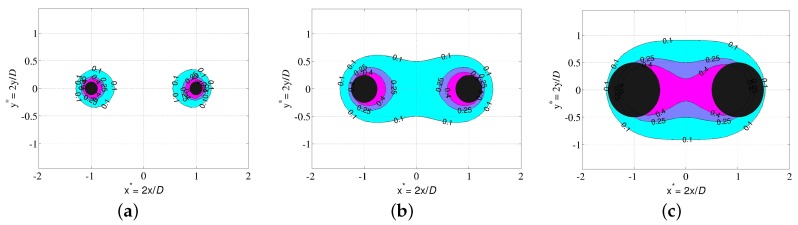
Cross sections in the dimensionless plane $x^{*}=2\;x/D$ $y^{*}=2\;y/D$ of the spatial distribution of sensor sensitivity in medium. Distribution is deduced from the expression of electrical field for the electrode design in Figure 8. Each contour corresponds to points in which sensitivity normalized by its maximum value has a value η, with η=0.40 (filled with magenta color), η=0.25 (purple) and η=0.10 (blue), respectively. The distribution depends on the sensor geometry factor α=Φ/D, where *D* is the electrode spacing and Φ their diameter. Distributions are reported for (**a**) α=0.12; (**b**) α=0.25; (**c**) α=0.50.

On the other hand, the ratio α=Φ/D is important to fix the spatial distribution of sensor sensitivity around electrodes, hence the medium volume sampled by the sensor. Figure 9 shows the evolution with α of the cross sections of the sensitivity distribution, according to different cutoffs. Distribution is obtained from the analytic expression of the electric field produced by the sensor, assuming a homogeneous medium. Results were validated by measurements of sensitivity profiles of a multi-channel sensor in air, using a waver-filled tube placed at different points (details in a submitted article). Electrodes too far from each other, or low α, produce a sample volume localized around electrodes with a thickness fixed by their diameter. Close electrodes concentrate the sample volume in the space between them, somewhat like a plane capacitor. The volume is better defined, albeit small. A ratio α between 0.30 and 0.40 should represent a good compromise. In any case, a large volume depends on electrodes having a sufficiently large diameter relative to pore size encountered in the medium (Φ at least 6 mm).

### 2.4. Other Capabilities of the Sensor

**Figure 10:**
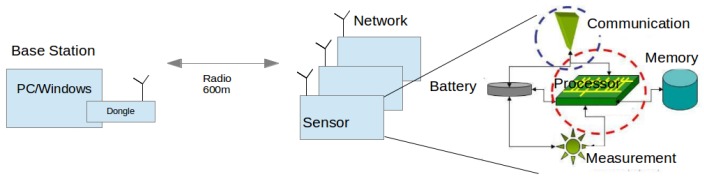
Schematic view of a wireless network of autonomous sensors as developed in our laboratory.

In addition to the bridge circuit, the sensor also comprises a digital part which performs the acquisition, treatment and transfer of bridge data. It thus plays the role of the data-logger. It also includes temperature measurement and its acquisition. The system developed for the sensors in Figure 1a,b was presented in a recent article [11], and only its main features are recalled. Figure 10 gives an overview of the different parts, from the processor in an autonomous wireless sensor to a computer that supervises a network of such sensors. Unlike the commercialized sensors presented in the introduction, the instrument analog circuit is closely integrated with the digital one, although each has its own board. All circuits are placed in the same waterproof housing, connected to electrodes in the medium via leads. Improvements of the system have been carried out for the last prototype (Figure 1d) in the area of transfer rate and energy autonomy. The prototype is also more compact and simple, using standardized components, for commercial purposes. Transfer rate has increased from 3.2 to 10 points·s^−1^ for a multi-channel sensor, and to 50 points·s^−1^ for a one-channel sensor, where a point comprises all data from all channels measured in an instant. Real-time monitoring is possible at a rate of one point every 10 min for the network or every 5 s for one sensor. This last possibility is rapidly limited by battery capacity (about 24 h with the new prototype). Consumption, including that for radio listening, in the absence of measurement amounts now to a power of 6 mW. The bridge requires a supply of 3.5 W, however, for a period shorter than 1 s every 10 min or more. A rechargeable Lithium-ion battery with a capacity of 45 Wh can provide a sensor with its energy for at least four months of autonomous operations.

The two first prototypes in Figure 1 dispose of a Pt thermometer placed inside one of the two electrodes of each channel to measure medium temperature at the same level as for permittivity. Simpler choices such as thermistors are studied for the next prototypes. Thermocouples are envisaged for multi-channel sensors in order to measure directly temperature differences between two channels. They can greatly increase the measurement accuracy and hence permit an assessment of heat flows.

## 3. Results and Discussion

**Figure 11:**
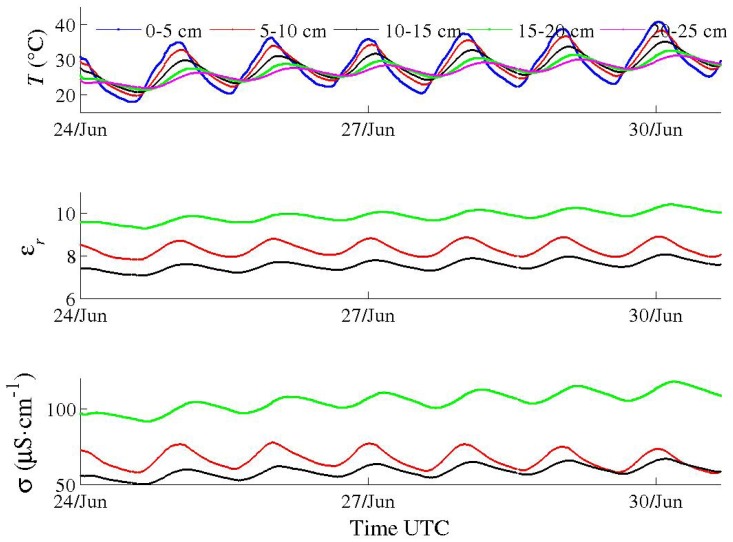
Time series of data recorded by a multi-channel sensor (*f* at 20 MHz) in sand with low clay content during summer 2015. Fluctuations of medium permittivity and conductivity of each channel are correlated to diurnal cycles of soil temperature at the same level. Data at depth 5–10 cm (red), at depth 10–15 cm (dark) and at depth 15–20 cm (green).

**Figure 12:**
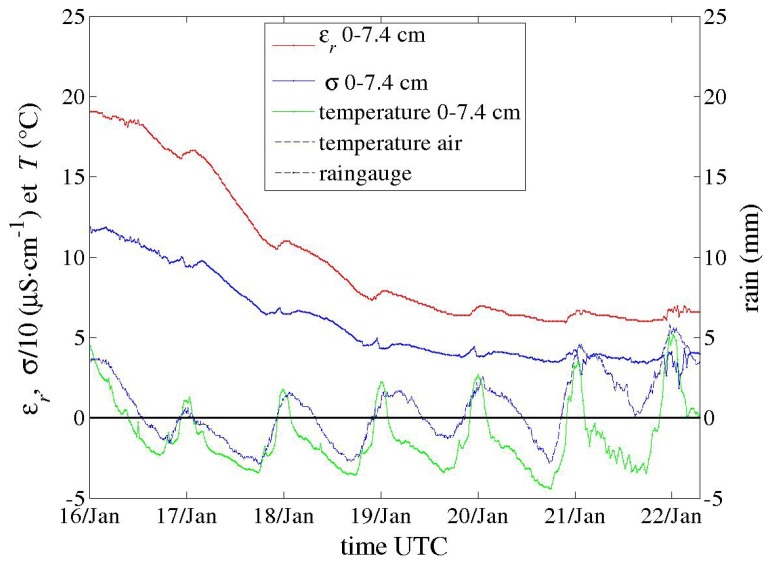
Time series recorded by a one-channel sensor (*f* at 10 MHz) during a cold spell in January 2017. Apparent medium permittivity and conductivity decreased towards low values as temperatures fell below 0 °C. Water in soil was progressively frozen. Rain and air temperature were provided by other sensors.

**Figure 13:**
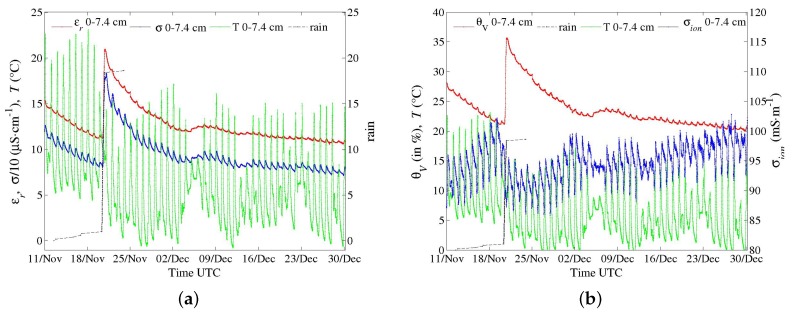
Series of data recorded by a one-channel sensor (Figure 1b; *f* at 20 MHz; a point every 10 min.) in a clayey calcareous soil at the end of 2015. (**a**) apparent medium permittivity, conductivity and temperature along with data from a rain gauge; (**b**) conversion into water content and salinity (see text).

Field trials have been performed for the last two years with the first prototypes (Figure 1a,b), primarily to test materials in various conditions for periods longer than one month. In spite of their large electrode diameter, multi-channel sensors were successively installed, thanks to their dedicated equipment, in hard soils such as a silty sand with a 10% clay content (Illite and Kaolinite) and a clayey calcareous soil. Problems of electric contact at the electrode level, which arose from differential thermal contractions of materials, were later solved. Energy consumption of circuits in these first prototypes were deemed too high. However, long time series in meteorological conditions from a hot summer and a frost were recorded, such as those shown in Figure 11, Figure 12 and Figure 13. In Figure 12, apparent medium permittivity and conductivity decreased towards low values as temperatures fell below 0 °C and water in soil was progressively frozen.

**Figure 14:**
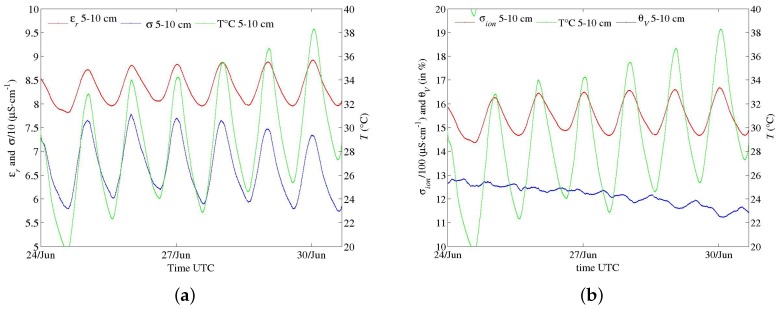
Data from Figure 11 for the channel at depth 5–10 cm. (**a**) apparent medium permittivity, conductivity and temperature; (**b**) conversion into water content and salinity (see text).

These measurements make evident the influence of diurnal cycles of temperature in soil on conductivity σ and real permittivity $\varepsilon_{r}$, especially during a dry and hot summer (Figure 11). The fluctuations are so well synchronized with temperature ones at each depth that any bias due to electronics sensitivity is ruled out. The correlation of apparent permittivity with medium temperature is positive (with $\alpha_{\varepsilon_{r}}$ about +0.8% °C^−1^ from the cycle of the 27 of June in Figure 14), as opposed to the behaviour of the water permittivity ($\alpha_{\varepsilon_{r_{w}}}=-0.46\%$ °C^−1^ at 25 °C [27]). Hence, the main mechanism is not related to the polarization of water molecules (or to the thermal dilation of the volume, being negligible: 0.026% °C^−1^ at $T_{0}=25$ °C). The phenomenon has been already observed [28,29]. One explanation attributes it to the water bound to particle surface, of which dipoles have a relaxation frequency lower than that of free water dipoles [30], even lower than *f* of sensors (they operate here at f=20 MHz). Bound water permittivity is then close to the silicate one, about 5, and, therefore, the water content as measured by sensors does not include bound water. However, its relaxation frequency increases with the temperature and can become higher than *f*. Hence, permittivity of bound water increases dramatically and its content is measured.

**Figure 15:**
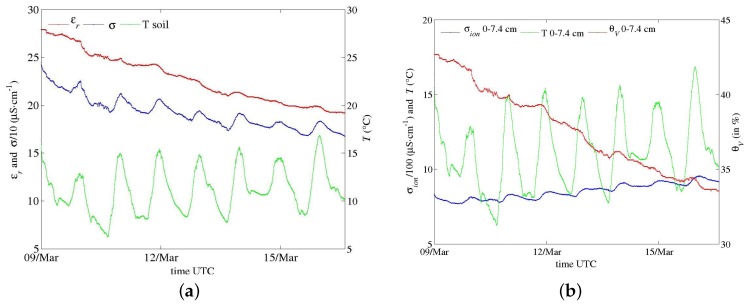
Time series recorded by a one-channel sensor (*f* at 20 MHz) in the same soil as in Figure 11 and Figure 14, just after a large amount of rain. (**a**) apparent medium permittivity, conductivity and temperature; (**b**) conversion into water content and salinity (see text).

**Figure 16:**
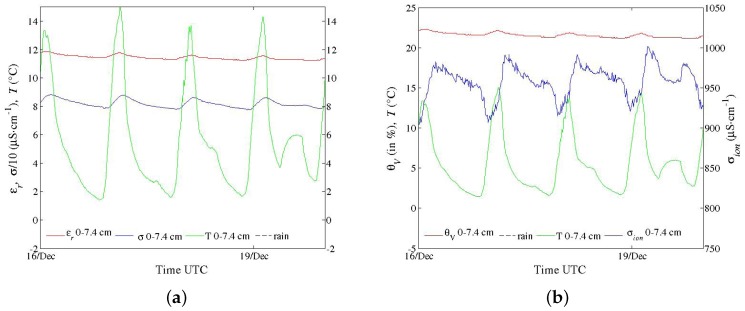
Zoom in on the series in Figure 13. (**a**) apparent medium permittivity, conductivity and temperature; (**b**) conversion into water content and salinity (see text).

This effect is more acute for drier soil with a low amount of free water, as observed in Figure 14 versus Figure 15. The general effect is also observed in the calcareous soil (Figure 13 and Figure 16), albeit weak as the soil remains wet. An in-depth analysis would require a thorough study in a controlled environment, which is not within the scope of the present article.

Figures of σ and $\varepsilon_{r}$ series are paired with ones of soil water content $\theta_{v}$ and its salinity $\sigma_{ion}$ after conversions. $\theta_{v}$ is deduced from $\varepsilon_{r}$ thanks to Topp’s correlation, as a first attempt. Because of its simplicity, the relation does not correct for the temperature dependence. As far as $\sigma_{ion}$ is concerned, Hilhorst, in the case of sensors measuring simultaneously σ and $\varepsilon_{r}$, and therefore in same conditions of geometry and frequency—-as in the case of our sensors—, suggested the following relation [2,31]:

(11) $$\sigma_{ion}=\frac{\sigma \;\varepsilon_{r_{w}}}{\varepsilon_{r}-\varepsilon_{r(\sigma =0)}},$$

where $\varepsilon_{r_{w}}$ is the relative permittivity of pure water and $\varepsilon_{r(\sigma =0)}$ medium permittivity towards σ=0. The latter corresponds approximately to the dried medium. The default value suggested by Hilhorst, $\varepsilon_{r(\sigma =0)}$ = 4.1, is used. As regards the fluctuations of σ, the positive correlation with temperature could be explained by the dependence of mobility of ions in pore water, or $\sigma_{ion}$, with a coefficient $\alpha_{\sigma_{ion}}$ between +2.0 and +2.5% °C^−1^ [27]. However, the time series obtained from Equation (11) do not agree with this established dependence, especially in a dry soil where $\alpha_{\sigma_{ion}}$ seems close to 0 (Figure 14b). The expression is valid only for free water, which may explain this discrepancy. Moreover, the term $\varepsilon_{r(\sigma =0)}$ may also depend on the temperature. As for permittivity, further investigations are necessary but are beyond scope of the present article.

## 4. Conclusions

The article has presented new sensors and their performance to measure continuously and accurately in situ intrinsic quantities of a porous medium such as its water content, the water salinity and its temperature. The measurement principle is based on the determination of medium permittivity $\varepsilon_{r}$ and conductivity σ. It is carried out with a high resolution and speed by a self-balanced bridge, which measures the admittance formed by the sensor electrodes in the medium. All potential bias encountered in this type of instrument from electronic imperfections in the bridge to probe inductance are taken into account by a physical model to obtain an accuracy of about $\delta \sigma =\pm \left[1+1\%\;\sigma \right]\;\mathrm{mS}\cdot m^{-1}$ and $\delta \varepsilon_{r}=\pm \left[0.1+1\%\;\varepsilon_{r}\right]$, up to $\sigma =1\;S\cdot m^{-1}$. Dependence of sensitivity distribution around electrodes on their dimensions was also studied to optimize the medium volume sampled.

Sensor capabilities of autonomy and wireless communication have been also developed. Energy capacity and storage can assure continuous operations for at least three months, depending on frequency of measurement and data transfer.

Results of some field trials are presented. In particular, they make evident the influence of medium temperature on its permittivity and conductivity, which is not well captured by common models.

Design is also simplified and standardized to provide cost-effective sensors, while remaining robust and accurate, for hydrology studies or for economic activities.
